# Development of Starch-Based Materials Using Current Modification Techniques and Their Applications: A Review

**DOI:** 10.3390/molecules26226880

**Published:** 2021-11-15

**Authors:** Sumedha M. Amaraweera, Chamila Gunathilake, Oneesha H. P. Gunawardene, Nimasha M. L. Fernando, Drashana B. Wanninayaka, Rohan S. Dassanayake, Suranga M. Rajapaksha, Asanga Manamperi, Chakrawarthige A. N. Fernando, Asela K. Kulatunga, Aruna Manipura

**Affiliations:** 1 Department of Manufacturing and Industrial Engineering, Faculty of Engineering, University of Peradeniya, Peradeniya 20400, Sri Lanka; sumedha.sma@gmail.com (S.M.A.); nimashamf@gmail.com (N.M.L.F.); aselakk@eng.pdn.ac.lk (A.K.K.); 2 Department of Chemical and Process Engineering, Faculty of Engineering, University of Peradeniya, Peradeniya 20400, Sri Lanka; hishendri1995@gmail.com (O.H.P.G.); darshanawanninayaka1992@gmail.com (D.B.W.); amanipura@yahoo.com (A.M.); 3 Department of Material & Nanoscience Technology, Faculty of Technology, Wayamba University of Sri Lanka, Kuliyapitiya 60200, Sri Lanka; canfernando9@gmail.com; 4 Department of Biosystems Technology, Faculty of Technology, University of Sri Jayewardenepura, Homagama 10200, Sri Lanka; 5 Department of Materials and Mechanical Technology, Faculty of Technology, University of Sri Jayewardenepura, Homagama 10200, Sri Lanka; suranga@sjp.ac.lk; 6 Materials Engineering Department, California Polytechnic State University, San Luis Obispo, CA 93407, USA; wmanampe@calpoly.edu

**Keywords:** starch, chemical methods, biodegradable polymer, starch modification

## Abstract

Starch is one of the most common biodegradable polymers found in nature, and it is widely utilized in the food and beverage, bioplastic industry, paper industry, textile, and biofuel industries. Starch has received significant attention due to its environmental benignity, easy fabrication, relative abundance, non-toxicity, and biodegradability. However, native starch cannot be directly used due to its poor thermo-mechanical properties and higher water absorptivity. Therefore, native starch needs to be modified before its use. Major starch modification techniques include genetic, enzymatic, physical, and chemical. Among those, chemical modification techniques are widely employed in industries. This review presents comprehensive coverage of chemical starch modification techniques and genetic, enzymatic, and physical methods developed over the past few years. In addition, the current applications of chemically modified starch in the fields of packaging, adhesives, pharmaceuticals, agriculture, superabsorbent and wastewater treatment have also been discussed.

## 1. Introduction

**Figure 1:**
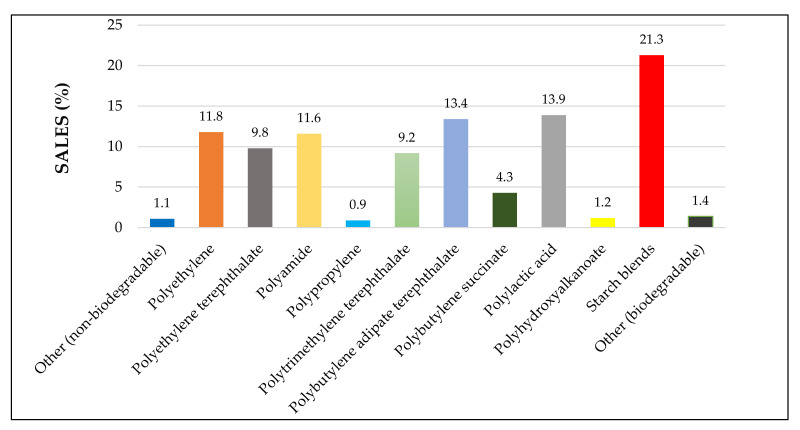
Global production capacity of different materials in 2019 (Adapted from European bioplastic data—2019 [6]).

Starch is a natural biopolymer extracted from plant sources and is a principal component in food formulations. It provides energy to humans by releasing glucose during cellular respiration [1]. Starch is also utilized in various applications, including food, polymer, paper, textile, laundry finishing products, and biofuel productions [2]. Due to the environmental impact caused by petroleum-based plastics, biopolymer-based plastics or bioplastics have been widely considered a sustainable alternative. The most commonly used bioplastics are starch blends, polylactic acid (PLA), polyhydroxyalkanoates (PHA), polybutylene succinate (PBS), poly (butylene adipate-*co*-terephthalate) (PBAT,) and polytrimethylene terephthalate (PTT). Among those many different biopolymers, starch possesses intriguing characteristics such as its relative abundance, low cost, biodegradability, surface functionalization, renewability, and non-toxicity [3]. According to the European bioplastic market data (2019), starch could fulfil about 21.3% of global biopolymer requirement in 2019, indicating its high demand over the recent years; see Figure 1.

Starch is mainly composed of amylose (80%), amylopectin (20%), lipid, protein (0.6%), and a small quantity of minerals (<0.4%) [4]. The amount of amylose and amylopectin varies according to the source of starch [3]. Amylose is insoluble in water, whereas amylopectin is soluble in water [3]. Starch also contains a small fraction of compounds known as “intermediate compounds,” which have intermediate properties of both amylose and amylopectin [5]. The molecular weight of amylose is around 10^5^–10^7^ Da, while amylopectin has a molecular weight of 10^7^–10^9^ Da. Moreover, the molecular weight of the intermediate compound is less than amylopectin and higher than amylose [5].

**Figure 2:**
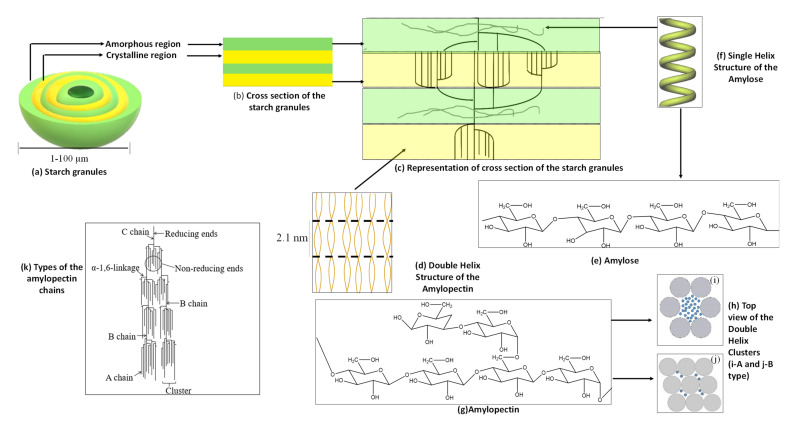
Schematic representation of the (**a**) Starch granules; (**b**) Cross section of the starch granule; (**c**) Representation of the cross section of the starch granules; (**d**) Double helix structure of amylopectin; (**e**) Structure of amylose; (**f**) Single helix structure of amylose; (**g**) Structure of amylopectin; (**h**) Top view of the double helix clusters: (**i**) A type, (**j**) B type, and (**k**) Types of amylopectin chains.

Starch granules are made of alternating layers of the amorphous and crystalline lamellae with thicknesses between 100 and 400 nm [7]. The starch granules have a density of about 1.5 g/cm^3^. The diameter of the granules generally ranges from 1 to 100 μm (see Figure 2a), and the shapes can be regular such as spherical, oval, angular, or quite irregular [3,8]. Moreover, the shape, size, structure, and composition of starch granules are dependent on their botanical source [4]. However, starch granules are not soluble in either cold water or water at room temperature due to the strong hydrogen bonds between the starch chains [7,9]. There are alternating layers of the crystalline and amorphous lamella, as shown in Figure 2b. The crystalline lamella of starch granules consists of double-helical amylopectin side-chain clusters, while amorphous lamella consists of amylopectin branching regions and amylose chains [3], as can be seen in Figure 2c,d. Amylose is composed of d-glucose units linked by α-1,4-glycosidic bonds and consists of small branches, as shown in Figure 2e [10,11]. Usually, amylose forms a single helical complex (see Figure 2f) in the presence of complexing agents [7,11]. Amylopectin is composed of α-d- glucose units linked by α-1,4 and α-1,6-glycosidic bonds (See Figure 2g) [3]. The linear amylopectin chains are relatively shorter compared to amylose chains. Moreover, amylopectin is highly branched compared to amylose [3]. Furthermore, the degree of polymerization (DP) of those external chains is around 10–20 as two chains unite into a double-helix with six glucose units per turn of each strand and a pitch of 2.1 nm, as shown in Figure 2d [11]. The length of these double-helices is about ~4–6 nm and crystallizes either into one of the two polymorphs, called A- or B-types [11]. A- type is known as the monoclinic unit cell with eight (8) water molecules, while B- type is a hexagonal unit cell with thirty-six (36) water molecules [3], as shown in Figure 2h,i and 2h,j.

The amylopectin structure contains three types of chains known as A, B, and C, as illustrated in Figure 2k [3]. The A-chain is linked to the α-d-glucose units only by a reducing end without bonding to other chains. The B-chains are bonded to either A-chain or other A- or B- chains via one or more hydroxyl groups (-OH) present in the amylopectin chains. The reducing end groups generally are carried by the C types of the chain [3].

The physicochemical properties of starch mainly depend on the amylose and amylopectin ratio [12,13,14]. The glass transition temperature (Tg) is one of the physical properties of the polysaccharides, which describes the temperature that transforms from an amorphous state to a viscous state [4]. At Tg, polymer material transits from a glassy, brittle state to a soft and flexible state. Since starch consists of both amorphous and crystalline regions, Tg of starch cannot be detected easily [4].

Native starch is not suitable for engineering applications due to its brittleness. Therefore, it requires a suitable plasticizer [15,16,17]. Although native starch is considered a non-plasticized material due to the presence of intra-and intermolecular -H bonding between the hydroxyl groups of starch molecules, starch can be converted into a continuous polymeric entangled phase when mixed with an aqueous or non-aqueous plasticizer such as glycerol, ethanolamine, urea, formamide, glycol, xylitol, sorbitol, sugars, and acetamide. When starch is plasticized, a semi-crystalline granule of starch is transformed into a homogeneous material due to the cleavage of hydrogen bonds between starch molecules. This behavior leads to a loss of crystallinity and lowers the glass transition temperature of starch while improving the chain flexibility. Finally, the plasticized starch becomes a petroleum-like thermoplastic starch (TPS) that is easy to process and moldable [16,18,19].

Glycerol is considered the most widely utilized plasticizer among various available plasticizers due to its non-toxicity, low cost, and high boiling point. Interestingly, the properties of TPS depend on the amount and the type of plasticizer added [16,19]. According to the literature, both modified starch and native starch can be used to prepare TPS. For example, Ahmed et al. [16] prepared TPS via dialdehyde starch, demonstrating reduced Tg, water absorptivity, hydrophilicity, and water vapor permeability with enhanced mechanical properties [16].

The semi-crystalline nature of starch is determined by the amylopectin component [20]. Water absorption and subsequent swelling of starch granule result in amylopectin-amylose phase separation and crystallinity loss [4]. Starch can also be solubilized using excess water at high temperatures (above 80 °C) by the depolymerization process [21]. Furthermore, starch solubilization can be enhanced using microwave, autoclave, and a boiling water bath [22,23,24,25]. Starch possesses an excellent film-forming characteristic. However, the higher water absorption and low mechanical strength [1] limit starch-based materials for engineering applications. Native starch contains many hydrophilic -OH groups resulting in moisture sensitivity. Due to amorphous regions, starch exhibits low mechanical properties, such as tensile strength, elongation at break, and brittleness [1].

**Figure 3:**
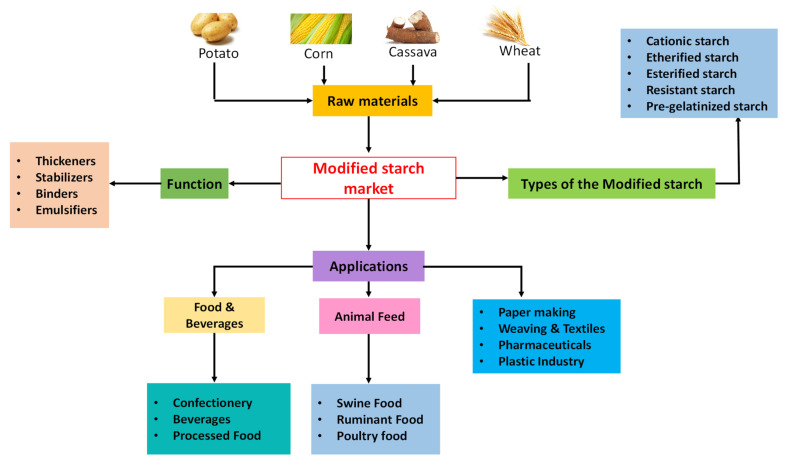
Modified starch market share by the application (Reproduced from: Modified starch market size, by product 2014–2025 (USD billion) Grand View Research, Inc. [27]).

In order to improve the mechanical properties of TPS, numerous modification techniques have been employed [26]. Figure 3 shows the raw materials, types of modified starch, their functions, and applications available in the market. 

The modified starch is mainly applied in the food, paper and pulp, textiles, and pharmaceutical industries [27]. In addition, a small percentage of modified starch is also used in the bioplastic industry. Common starch modification methods employed in the industry include heat–moisture treatment, annealing, retrogradation, freezing, ultra-high-pressure treatment, glow discharge plasma treatment, genetic modifications, enzymatic reactions, osmotic-pressure treatment, thermal inhibition, gelatinization, etherification, esterification, crosslinking, acid treatment, oxidation, and dual modification. However, those aforementioned starch modification methods can be categorized into four main processes: Genetic, enzymatic, physical, and chemical [26].

Several articles have recently been published on environmentally friendly or green starch modification techniques. For example, Fan and Picchioni [28] reported the newly emerging green solvent for the modification of starch, whereas Guida et al. [29] studied the green techniques available for starch modification to stabilize Pickering emulsions. Furthermore, Maniglia and coworkers [30] reviewed “clean” or “green” alternatives for starch modification and their possible applications. Moreover, Hj. Latip et al. [31] presented an overview, including the current investigations on the properties of starch produced via esterification/crosslinking with citric acid.

The current review focuses on the emerging starch modification techniques, including enzymatic, physical, and chemical for developing starch-based materials reported in the literature over the past few years. This review mainly discusses the chemical modification techniques of starch and their effect on the thermomechanical properties. In addition, a brief overview of genetic, enzymatic, and physical modification of starch and their main applications is also provided. Finally, the potential applications of the chemically modified starch in packaging, adhesives, pharmaceutical, agriculture, super-absorbents and wastewater treatment are also discussed in this review.

## 2. Starch Modification Processes

The following section discusses the four industrially important starch modification techniques with the main focus on chemical processes.

### 2.1. Genetic Modifications

Genetic modification of starch is a technique that involves transgenic technology. Genetic modification can be carried out using traditional plant breeding techniques or biotechnology. This technique uses the enzymes involved in starch biosynthesis [32]. Generally, plants, animals, and organisms, including bacteria and fungi, contain genes for enzymes that engage in starch biosynthesis. These genes can be used to produce genetically modified microorganisms, which produce those enzymes for starch modifications. For example, modified starch with amylose-free, high amylose content, or waxy rice starch consists of 0 to 3% of amylose can be produced via genetic modification processes [33].

Amylose-free starch has widely been tested as water binders, viscosity builders, and texturizers, mainly in food and other industrial applications. However, this starch is less resistant to shear, acid, and high temperatures than native starch, forming cohesive pastes with extended cooking [32]. High-amylose starch in cereals is produced via a genetic mutation of the gene that encodes the starch-branching enzyme, known as the amylose extender (ae) mutant. This ae mutant lacks the starch branching enzyme, resulting in longer amylose chains. Starch with high amylose content is an essential precursor for developing polymer coatings that are resistant to enzymatic degradation. Moreover, these coatings can also be used in colon cancer targeted drug delivery systems [34].

Bull et al. [35] investigated the effect of breeding techniques in transgene-free progeny cassava plants. The authors reported that the potential use of genetically modified starch varieties in food applications. Furthermore, Zhao et al. [36] prepared genetically modified potatoes with high cellulose, amylose, and less pectin. Moreover, Li et al. [37] explored the effect of the transgenic technique on the bioavailable vitamin B6 in cassava starch.

### 2.2. Enzymatic Process

During enzymatic modification, starch is exposed to enzymes (hydratases) that accelerate the production of highly functional derivatives [38]. The enzymatic modification technique is mainly used to prepare glucose syrup, high fructose corn, or cassava syrup. The primary function of enzymatic modification is the depolymerization of starch into oligosaccharides or the transformation of starch by transferring glycosidic linkages and residues [38]. The role of the enzyme during this modification technique is breaking down the α-1,4 bond between two glucose units [32]. Enzymatically modified starch is utilized in foodstuffs, cosmetics, pharmaceuticals, detergents, adhesives, and drilling fluids [32]. The main four types of starch converting enzymes are (i) endoamylses, (ii) exoamylases, (iii) debranching enzymes, and (iv) transferases [32]. Table 1 summarizes the examples of starch converting enzymes and their biological function.

**Figure 4:**
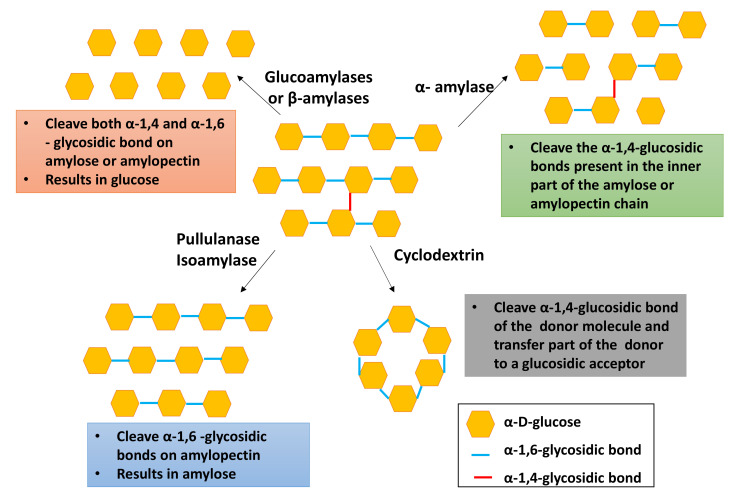
Enzymatic hydrolysis of starch.

Among the four types of starch converting enzymes, debranching enzymes are widely employed in the industry. Figure 4 demonstrates the enzymatic hydrolysis of starch. As depicted in Figure 4, pullulanase, isoamylase, and oligo-1,6-glucosidase are mainly used to debranch the α-1,6-glycosidic bonds in starch, giving linear amylose, whereas amylosucrase, α-amylase, and amylomaltase are capable of debranching the 1,4 and 1,6 glycosidic bonds, resulting in small amylose or amylopectin chains.

Table 2 summarizes the optimum reaction conditions for starch debranching enzymes. According to Figure 4, exoamylases such as glucoamylases, α-glucosidases, and β-amylases are used to produce glucose, whereas cyclodextrin cleaves the α-1,4-glycosidic bond while connecting both reducing and nonreducing ends. The isoamylase enzyme has higher enzymatic activity compared to that of other enzymes [38]. Pullulanase is widely employed to prepare high-amylose starch utilized in adhesive products, resistance starch, corrugated boards, and the paper industry [38].

Besides the previously mentioned enzymes, lipase and protease are also used for starch modification [41]. These enzymes are widely utilized to catalyze starch acetylation and achieve modified starch with high purity [42]. Gill et al. [43] reported the green synthesis of corn starch-maleate using the lipase enzyme as a biocatalyst and maleic acid as an esterification agent. Adak et al. [44] prepared esterified starch using lipase and ionic liquid.

### 2.3. Physical Modifications

The physical properties of the starch can be modified using physical processes. Physical processes are generally divided into thermal and non-thermal treatments [45]. Table 3 summarizes the physical starch modification techniques and their basic features.

Usually, physically modified starch is considered natural as opposed to chemically modified starch [45]. Physical modifications are more accessible and often less expensive than chemical modification techniques and produce no effluents containing salts, reagents, or reagent by-products [46]. In general, physical treatments destroy the arrangements of the starch molecules within granules. Even though the properties of physically treated starch are the same as in native starch, they are not as thermostable as the starch obtained through chemical modification [47]. Thermal treatment, including annealing, microwave radiation, pre-gelatinization, and heat–moisture treatment (HMT) [48], are the most widely applied physical processes, as shown in Table 3. The microwave-assisted treatment is commonly applied during the preparation of films from starch. Generally, microwave-assistant treatment increases water solubility and decreases the crystallinity, viscosity, and transparency of starch [49]. In addition, the microwave-assisted treatment has several advantages such as energy-saving, high conversion, and rapidity [50]. Souza et al. [51] prepared cassava starch-based biodegradable films using the microwave-assistant method. The authors also reported the use of clay nanoparticles as a reinforcement material to develop cassava starch-based biodegradable packaging materials. Zhao and coworkers investigated the effectiveness of the combination of microwave treatment and chemical modification techniques. The authors reported improvements in the degree of substitution, structural, and physicochemical properties of potato starch esters obtained by microwave pretreated esterification [52].

**Table 3:** A summary of physical starch modification processes.

Physical Modification		Description	References
Thermal treatments	1. Pre-gelatinization	Results in instant starch Heat and dry under gelatinized conditions without allowing molecular re-association	[53]
	2. Heat Moisture Treatment (HMT)	A hydrothermal process Heat starch granules in a sealed vessel at a temperature above the glass transition temperature of starch. Strengthens starch granules under higher temperatures and low moisture environment	[54]
	3. Annealing	A hydrothermal process Hold starch granules in excess water at a temperature between the T_g_ (25 °C) and gelatinization temperature of starch (100 °C)	[53]
	4. Microwave (MV) heating	Materials absorb microwave energy, and the energy is converted into heat through molecular vibration and friction	[55]
Non-thermal treatments	1. Milling	Use the mechanical force to change the starch properties Alter granule morphology, crystallinity, solubility, and swelling properties	[56]
	2. High-Pressure Treatment (HPT)	Two types: (i) Static type or Ultra High-Pressure (UHP) and (ii) High-Hydrostatic Pressure (HHP) treatment Use homogenizers to produce turbulence, high shear, and cavitation in starch slurry through the high pressure	[56]

Oyeyinka et al. [57] studied the effect of microwave treatment on the structure, surface, and porous nature of starch granules. For example, the pores created during microwave treatment may facilitate the movement of plasticizers and other additives added to the starch granules. The authors also investigated the effect of microwave radiation on the physicochemical properties of the cassava starch-stearic acid complex. This work demonstrated that microwave radiation improved the complexation of stearic acid with cassava starch, giving high thermal stability and viscosity. Importantly, physical modification via microwave treatment is relatively faster over conventional thermal treatments [58].

Xie et al. [55] explored the molecular weight of native potato starch upon microwave treatment. The authors showed that the native potato starch with a molecular weight of 7.62 × 10^7^ reduced down to 7.25 × 10^7^, 5.42 × 10^7^, 4.59 × 10^7^, and 1.72 × 10^7^ (Da) after 5, 10, 15, and 20 s of microwave treatment, respectively. Furthermore, Lewandowicz et al. [59] found that the change in the crystal structure of potato starch from type B to type A during microwave treatment. Therefore, it is conclusive that microwave radiation has a significant impact on the structural properties of starch.

### 2.4. Chemical Modifications

**Figure 5:**
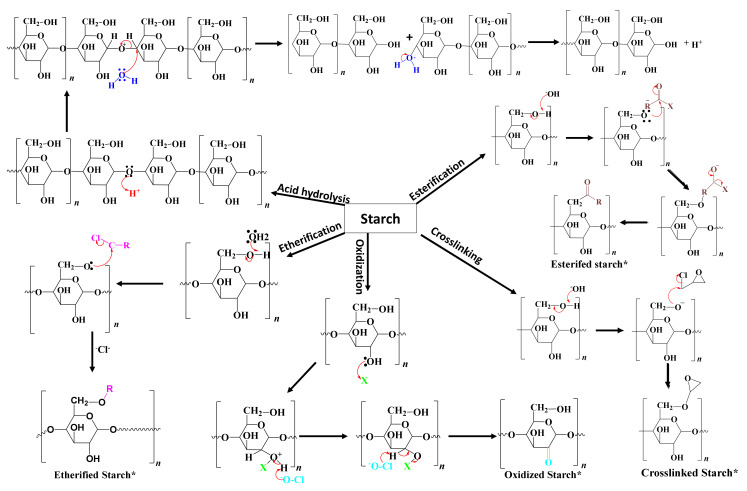
The reaction mechanisms of different chemical modification processes.

Chemical modification involves the introduction of functional groups into the starch molecules. The chemical modification techniques depend on the starch source, reaction conditions such as concentration, reaction time, pH, the presence of a catalyst, and the type of substituent [60]. Examples of chemical modification processes are etherification, esterification, crosslinking, grafting, and decomposition (acid or enzymatic hydrolysis) [1]. However, the key issue with chemical modification is the generation of toxic residues. Figure 5 shows the reaction mechanisms of different types of chemical modification processes. The following section provides a comprehensive description of different chemical processes commonly used for starch modification and their applications.

#### 2.4.1. Crosslinking

**Figure 6:**
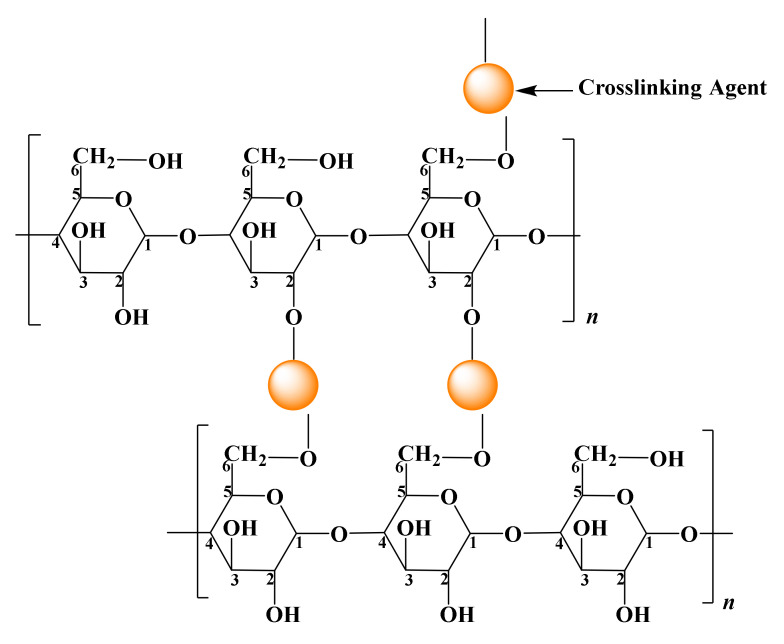
Schematic of crosslinked starch.

Crosslinking of starch is the reaction between the hydroxyl groups of the starch molecule and a compound with two or more functional groups [61]. A crosslink is an ionic or covalent bond that connects one starch chain to another. Crosslinking agents bridge the adjacent starch chains [9]. For example, a bifunctional or multifunctional crosslinking agent forms an intermolecular linkage with the primary hydroxyl groups at C2 and C3 carbons and the secondary hydroxyl group at C6 carbon [60], as shown in Figure 6.

**Figure 7:**
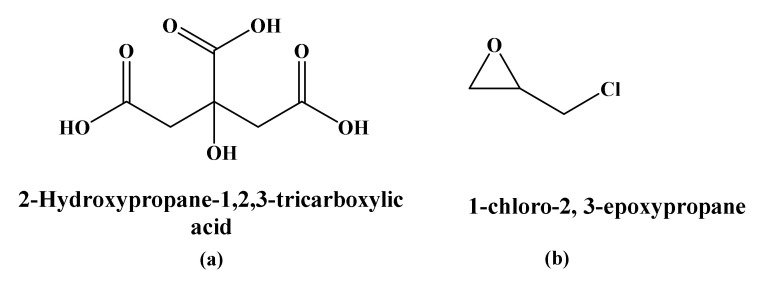
Chemical structures of crosslinking agents: (**a**) Citric acid and (**b**) epichlorohydrin.

Widely used crosslinking agents are citric acid, glutaraldehyde, phosphorus oxychloride, sodium trimetaphosphate (STMP), sodium tripolyphosphate (STPP), and epichlorohydrin (EPI) [9,60]. Crosslinked starch has remarkable properties compared to native starch, such as high mechanical, thermal properties, and water stability, as shown in Table 4. Citric acid (see Figure 7a) is one of the commonly tested crosslinking agents, which forms a strong hydrogen bond with starch, giving improved thermal and water stabilities and inhibiting retrogradation [62]. For example, Reddy and Yang used citric acid as a crosslinker for corn starch and reported increased water and acid stabilities (see Table 4) [61]. Seligra et al. [63] conducted a study to explore the effect of citric acid on the biodegradable and non-retrograde starch-glycerol-based films. This work indicated that the addition of citric acid as a crosslinking agent decreased the water vapor permeability (WVP) by 35.71% while avoiding starch retrogradation and maintaining degradability during composting. Such observations were ascribed to the substitution of hydrophilic -OH groups by the hydrophobic ester groups. In addition, citric acid has also been used as an antibacterial agent.

Epichlorohydrin (EPI), also known as 2-((chloromethyl)oxirane), is an organochlorine compound, see Figure 7b. The reaction of EPI with starch is considered a multifunctional reaction since one or two EPI molecules react to produce only one crosslinked bond (glycerol bond). However, starch crosslinked with EPI is not homogeneous [64]. Rioux et al. showed the improved tensile strength of high amylose corn starch thin films [65]. Carmona-Garcia and coworkers exhibited a decrease in the moisture absorbance for banana starch upon crosslinked with EPI [64], as shown in Table 4.

The blending of starch films with other synthetic or natural polymers is another common technique to improve the mechanical properties of starch. During mixing, EPI is used as a crosslinking agent. For example, potato starch-polyethene blended films, which were crosslinked with EPI, showed increased tensile strength and elongation at break of 54.48% and 8.32%, respectively [66]. Detduangchan et al. [67] displayed enhanced tensile strength of the starch thin films due to crosslinking with EPI, STMP, and STMP/STPP, as depicted in Table 4.

Canisag [9] compared the mechanical properties of oxidized and crosslinked sucrose films with non-crosslinked films. Oxidized sucrose acted as a crosslinking agent due to the presence of aldehyde groups [9]. The corn starch films crosslinked with oxidized sucrose exhibited tensile strength of 22.9 MPa and 59.5% elongation at break [9]. EPI crosslinked rice starch films also showed superior water barrier properties compared to untreated rice starch films, as shown in Table 4 [67].

#### 2.4.2. Acid Hydrolysis

Acid hydrolysis is one of the most widely used chemical processes. Acids including sulfuric acid (H_2_SO_4_) and hydrochloric acid (HCl) are commonly applied in acid hydrolysis. Acid hydrolysis is an electrophilic substitution reaction that occurred in the presence of an electrophilic hydroxonium ion (H_3_O^+^) supplied by the acid [60]. Acid hydrolysis is an electrophilic substitution reaction that occurred in the presence of an electrophilic hydroxonium ion (H_3_O^+^) supplied by the acid [60]. Acid hydrolysis is initiated by attracting H_3_O^+^ molecules to the oxygen atom on the α-1,4-glycosidic bonds, as shown in Figure 5. The acid hydrolysis results in an increase in the crystallinity of starch [68]. During acid modification, amylopectin molecules are disbranched, increasing the linear component of the treated starch and the amylose-like behavior [69]. However, the key drawbacks of acid hydrolysis of starch include the random attack at the branch point, high glucose yield, and difficulty of removing excess acid [60]. Alternatively, the presence of long-chain alcohols reduces the degree of polymerization of amylopectin more effectively and converts the crystalline regions into more amorphous areas [70].

Sakkara et al. examined the effect of pH on the physicochemical properties of films developed from starch and showed that the mechanical and structural properties of starch were significantly affected upon changing the pH [71]. The authors reported that such behaviors could be attributed to the conversion of amylose into small amylose chains and glucose units during acid hydrolysis. Furthermore, these small amylose chains form strong inter-chain hydrogen bonding, improving the tensile and plasticity properties of modified starch [72].

Luchese et al. [73] developed the acid hydrolyzed pinhao starch films by varying the HCl acid concentration. They reported an increase in the water solubility of biofilms and a reduction in water vapor permeability (WVP). Usually, water vapor preferentially permeates through the amorphous areas. However, acid hydrolysis promotes fast hydrolysis of the amorphous regions over crystalline regions of starch, reducing the WVP. For example, Zhang et al. [72] reported that the WVP of the films prepared from acid hydrolysis was lower than native starch-based films, see Table 4. The authors also described the dependency of the WVP value of a polymer film on the number of polar sites in the polymer matrix. The WVP values increased with increasing polar sites due to the interaction between water molecules and surface polar groups.

#### 2.4.3. Alkali Treatment

Bases such as potassium hydroxide (KOH) and sodium hydroxide (NaOH) are widely used to modify starch. However, the alkali medium works more effectively on dry starch. After the addition of alkali, the mixture should be neutralized using hydrochloric acid or acetic acid.

Qin et al. [74] investigated the effect of alkali treatment on the properties of thermoplastic starch films prepared by melt extrusion. The data showed that increasing NaOH concentration changed the tensile strength of high amylose corn starch (HAS) and gradually increased the elongation at break. Moreover, the tensile strength of the HAS film with 2% of NaOH slightly increased up to 10.03 MPa (see Table 4), while its elongation at break also increased up to 40%. The increased tensile strength and elongation at break were attributed to the low alkali concentration promoting the gelatinization of HAS. The gelatinization facilitated the leaching of amylose from the crystalline structure while forming more hydrogen bonds with amorphous regions. Besides, NaOH also rearranged the molecular chain of HAS and changed the crystal structure of starch, increasing the space within molecular chains and improving the elongation at break. The HAS film treated with 10% NaOH exhibited the lowest tensile strength of 7.96 MPa and the highest elongation at break of 60%. During the melt extrusion process, NaOH oxidized the anhydroglucose of starch, causing irregular crosslinking of the molecular chains while increasing the degree of branching and molecular gap of the starch chain, resulting in increased flexibility and reduced tensile strength [74].

According to the research carried out by Sakkara et al. [71], the increase in pH reduced the tensile strength and the modulus while exhibiting an increase in the elongation at break. The highest elongation at break and the strength of the films were obtained at pH 11 and 5, respectively. Moreover, compared to the elongation at pH 7, the pH 11 films showed a significant increase in elongation at break. Furthermore, the elongation of the films varied from 1.9 to 2.5%, which was considerably lower than synthetic polymer-based films.

**Table 4:** Changes in the properties starch upon chemical modification.

Property of Starch	Chemical Modification		Values		References
	Reaction Type	Reagent	Before	After	
Viscosity	Oxidation	Sodium hypochlorite	173.8 mPa·s	151.8 mPa·s	[75]
	Grafting	Cassava starch grafted with Poly(acrylamide) (CS-g-PAM)	1717 mPa·s	4178 mPa·s	[76]
Tensile strength	Crosslinking	Epichlorohydrin (EPI)	10.04 MPa	15.51 MPa	[66]
	Crosslinking	Epichlorohydrin (EPI)	5.01 MPa	7.99 MPa	[67]
	Crosslinking	Sodium Trimetaphosphate (STMP)	5.01 MPa	8.23 MPa	[67]
	Crosslinking	Sodium Trimetaphosphate (STMP)/Sodium Tripolyphosphat (STPP)	5.01 MPa	7.57 MPa	[67]
	Alkali treatment	Sodium Hydroxide	9.51 MPa	10.03 MPa	[74]
	Etherification	Carboxymethyl starch	1.1 MPa	0.2 MPa	[77]
	Oxidation	Sodium hypochlorite	3.8 MPa	1.8 MPa	[75]
	Oxidation	Sodium hypochlorite	3.53 MPa	6.07 MPa	[78]
	Grafting	Thermoplastic Starch Grafted with Polylactic acid	2.40 MPa	0.06 MPa	[79]
	Oxidation	Sodium hypochlorite	3.88 MPa	5.05 MPa	[80]
	Oxidation	Sodium hypochlorite	4.66 MPa	8.39 MPa	[81]
	Oxidation	Hydrogen peroxide	18.8 MPa	24.7 MPa	[82]
	Etherification	Hydroxypropylated starch	3.1 MPa	4.9 MPa	[83]
	Grafting	Starch grafted with polystyrene	43 MPa	25 MPa	[84]
	Etherification	Carboxymethyl starch	31 MPa	14 MPa	[85]
Water vapour permeability	Crosslinking	Citric acid	33 g h^−1^·m^−2^	31 g·h^−1^·m^−2^	[61]
	Crosslinking	Citric acid	2.8 × 10^−10^ g/ms·Pa	1.8 × 10^−10^ g/ms·Pa	[63]
	Crosslinking	Epichlorohydrin (EPI)	6.19 g·mm/m^2^·day·KPa	1.89 g·mm/m^2^·day·KPa	[67]
	Crosslinking	Sodium Trimetaphosphate (STMP)	6.19 g·mm/m^2^·day·KPa	2.28 g·mm/m^2^·day·KPa	[67]
	Crosslinking	STMP/STPP	6.19 g·mm/m^2^·day·KPa	2.72 g·mm/m^2^·day·KPa	[67]
	Acid hydrolysis	Hydrochloric acid	3.16 × 10^−12^ g·cm·cm^−2^·s^−1^·Pa^−1^	5.74 × 10^−12^ g·cm·cm^−2^·s^−1^· Pa^−1^	[72]
	Acid hydrolysis	HCl (36%, w/w) at 45 °C for 24 h under stirring (225 rpm)	0.30 g·mm·day^−1^·m^−2^·mm·Hg^−1^	0.10 g·mm·day^−1^·m^−2^·mmHg^−1^	[73]
	Oxidation	Sodium hypochlorite	9.3 g·mm/m^2^·day·kPa	4.4 g·mm/m^2^·day·kPa	[75]
	Oxidation	Sodium hypochlorite	16.23 × 10^−11^ g·Pa^−1^· s^−1^·m^−1^	21.54 × 10^−11^ g·Pa^−1^·s^−1^·m^−1^	[80]
Elongation at break	Crosslinking	Epichlorohydrin (EPI)	200.42	217.11	[66]
	Etherification	Carboxymethyl starch	40%	140%	[77]
	Oxidation	Sodium hypochlorite	85.20%	84.90%	[78]
	Grafting	Thermoplastic Starch Grafted with Poly(lactic acid) (TPS-g-PLA)	68.00%	150.00%	[79]
	Oxidation	Sodium hypochlorite	17.91%	27.2%	[80]
	Oxidation	Hydrogen peroxide	650%	575%	[82]
	Oxidation	Hydroxypropylated starch	46.4%	43.5%	[83]
	Grafting	Poly(methyl methacrylate) grafted with modified Starch and styrene-butadiene rubber bio composites (PMMA-g-TPS/NR)	564%	888%	[86]
Thermal stability (Decomposition Temperature)	Esterification	Acetic anhydride	297 °C	352 °C	[87]
	Grafting	Corn starch grafted with Poly(methyl methacrylate) (CS-g-PMMA)	310 °C	332 °C	[88]
	Grafting	Starch grafted with *N*-tert-butylacrylamide	316 °C	343 °C	[89]
Moisture absorbance	Crosslinking	Citric acid	30%	20%	[65]
	Crosslinking	Phosphoryl chloride (POCl_3_)	6.72%	1.24%	[64]
	Crosslinking	Sodium Trimetaphosphate (STMP)	6.72%	3.29%	[64]
	Crosslinking	Epichlorohydrin (EPI)	6.72%	3.22%	[64]
Crystallinity	Crosslinking	Epichlorohydrin (EPI)	39.59%	38.11%	[66]
	Crosslinking	Epichlorohydrin (EPI)	16.10%	15.67%	[67]
	Crosslinking	Sodium Trimetaphosphate (STMP)	16.10%	15.13%	[67]
	Crosslinking	Sodium Trimetaphosphate (STMP)/Sodium Tripolyphosphat (STPP)	16.10%	14.79%	[67]

#### 2.4.4. Esterification

Esterification is the substitution of hydrophilic hydroxyl groups of starch by various hydrophobic groups, as shown in Figure 5. The degree of substitution is the number of hydroxyl groups replaced by the substituted groups per monomer unit [90], and the quality of the modified starch depends on the degree of substitution. The degree of substitution (DS) depends on the concentration of reagent, type of the reagent, catalyst, and duration of the reaction time. This method can be used to reduce the water absorption capacity of the starch. In starch, esterification weakens the intermolecular bonding that holds the granules together. As a result, it alters the granule shape, sizes, and other functional properties of the starch. The main types of esterification reactions are acetylation, succinylation, and phosphorylation. Among those, the acetylation reaction is widely used for esterification.

In acetylation, the starch reacts with acetic anhydride to produce acetylated starch. The hydroxyl group of the d-glucose units is esterified with the acetyl groups, incorporating acetate functionality [1]. Acetylation enhances the hydrophobicity of starch. It also increases the thermoplasticity, thermal stability, and film-forming ability of starch [87]. The acetate groups act as pegs on the amylopectin chains to prevent retrogradation. Films prepared from acetylated amylopectin films show reduced elongation at break. However, acetylation causes an increase in the crystallinity of amylopectin by densely packing the amylopectin branched structure [91]. The elasticity of starch acetyl films can be improved using external plasticizers such as glycerol [92].

#### 2.4.5. Etherification

Etherification of starch is usually achieved using epoxide reagents such as ethylene oxide. Initially, epoxides cleave the C-O bond under aqueous, acidic, or alcoholic conditions before the eventual condensation with starch, see Figure 5. Some etherification reactions occur under alkaline conditions [93]. Etherified starch is resistant to acid cleavage, alkali, and smooth oxidizing agents. The etherification of the OH group in starch leads to significant changes in their properties, avoiding the formation of starch aggregates and improving the physical properties [94]. Furthermore, starch etherification facilitates the gelatinization of starch [93].

Both esterification and etherification introduce lipophilic groups into the starch chains by reducing the hydrophilicity and the degree of inter-and intra-molecular hydrogen bonding [75]. However, the main advantage of etherification is to maintain starch stability by permitting the use of water-oil emulsion [95].

Carboxymethyl starch (CMS) is commonly used in the industry. The CMS is mainly produced due to its unique properties, such as high viscosity and stability [96]. During the CMS preparation, the linear and branched starch polymers are reacted with monochloroacetic acid (ClCH_2_CO_2_H) in an alkaline solution while adding carboxymethyl groups at the -OH positions of starch by ether linkages. Upon the addition of carboxymethyl groups, the starch becomes more resistant to thermal degradation and bacterial attack [96]. In this study, the authors reported a higher elongation at break (250% increment) for CMS-based films [96], as shown in Table 4. For instance, Wilpiszewska [77] prepared films using starch and carboxymethyl starch. The authors also showed a higher elongation at break with a 250% increment for CMS-based films, as shown in Table 4.

#### 2.4.6. Oxidation

In oxidation, the hydroxyl groups at C-2, C-3, and C-6 are oxidized to carbonyl groups and then to carboxyl groups. After that, starch is hydrolyzed to amylose and amylopectin molecules by breaking α-1,4-glycosidic linkages, as shown in Figure 5. The main oxidizing agents include periodate, chromic acid, permanganate, nitrogen dioxide, and sodium hypochlorite [97]. However, sodium hypochlorite is widely used as the oxidizing agent for starch modification in the industry [97]. The oxidization process is influenced by the molecular structure, origin, packing of the crystalline lamella, and the size of the amorphous lamella of starch [97].

For instance, Fonseca et al. [75] prepared biodegradable potato starch films using sodium hypochlorite as the oxidizing agent. According to their results, films made with oxidized starch exhibited lower tensile strength (1.8 MPa) than native starch films (3.8 MPa). However, these films showed lower water solubility with a 52% reduction of WVP compared to native starch films. García et al. [97] investigated the effect of zein (the protein found in maize starch) on native and oxidized banana starch along with different film formulations on mechanical and structural properties. The oxidized starch showed a 30% increase in tensile strength and a 53% increase of elongation at break compared to the native starch. However, these results did not agree with the values reported by Fonseca et al. [75]. Such deviation might be due to the variations in amylose and amylopectin ratios and variable oxidation conditions. The increased tensile strength of films was due to oxidized starch forming hydrogen bridges with the -OH groups of amylose and amylopectin molecules. These linkages provide more structural integrity in the polymeric matrix, thereby increasing tensile strength [78].

Zavareze et al. [78] developed potato starch-based films using sodium hypochlorite as the oxidation agent. The films produced from oxidized potato starch showed decreased solubility, elongation at break (0.3%), WVP (21%) values, and increased tensile strength compared to native starch films.

#### 2.4.7. Grafting

During grafting, synthetic or natural polymers are introduced to the backbone of the starch. The graft polymerization can tailor the chemical and physical properties of starch without changing the biodegradability [69]. The major sites for initiating graft copolymerization are localized at Cl-C2 end groups and C2-C3 glycol groups. Acrylate esters, methacrylate esters, and styrene are commonly used for graft polymerization. The hydrophobic grafted branches make starch less hydrophilic than unmodified starch. In addition, grafted starch is biodegradable and possesses higher tensile strength and a better appearance than unmodified starch [69]. However, the TPS grafted with polylactic acid exhibited lower tensile strength than native TPS, with the tensile strength of 2.40 MPa and 0.06 MPa for native and grafted TPS, respectively [79]. Even though the tensile strength of grafted starch decreased, the elongation at break increased by 120% [79]. This study also reported a lower Tg for grafted starch than native starch due to the role of grafted starch as an internal plasticizer, making micro-and macro-level changes [69]. Moreover, starch graft-copolymers with acrylate, methacrylate esters, or styrene are mainly used to prepare starch-filled plastics due to the compatibility of grafted starch with the plastic matrix [69].

The grafting reaction of starch is a free radical reaction and thus requires the treatment of initiators to yield free radical sites on the starch chain. After that, the starch chain acts as macro-initiators in the presence of synthetic monomers to obtain polymer grafts of high molecular weight [98]. Li et al. [88] prepared the cassava starch-poly (methyl methacrylate)-(CS-g-PMMA) copolymers. The results indicated that the presence of poly (methyl methacrylate) grafts improved the thermal stability of starch, and SEM micrographs exhibited a strongly deformed structure of CS-g-PMMA copolymer granules. The grafted starch also showed a higher thermal decomposition temperature of 332 °C than native corn starch.

#### 2.4.8. Dual Modification

Dual modification of starch involves combining either chemical and physical methods or chemical and enzymatic or genetic methods [99]. Woggum et al. [100] carried out a study to explore the properties of dual modified rice starch-based biodegradable films. The starch modification was conducted by hydroxypropylation with propylene oxide, followed by crosslinking with sodium trimetaphosphate (STMP). According to their results, the dual modified rice starch (DMRS) films showed increased tensile strength, elongation at break, film solubility, and decreased transparency value with increasing propylene oxide. In addition, XRD analysis of the DMRS films showed a decrease in crystallinity. Table 4 summarizes various chemical modification techniques and the respective changes in the physical properties of starch.

### 2.5. Applications of Modified Starch

The following section discusses the applications of enzymatically, physically, and chemically modified starch with the major focus on chemically modified starch.

#### 2.5.1. Applications of Enzymatically Modified Starch

Enzymatically modified starch is commonly used in food and non-food applications such as pharmaceuticals and drug delivery systems [101]. Enzymatically modified starch used in food products possesses enhanced properties, including softness, freshness, and shelf-life of baking products [102]. For example, Le Loan et al. [103] investigated the gluten-free rice bread prepared using β-amylase and d-enzyme. Moreover, Montoya and coworkers reported starch acylation via the B-lipase of Candida antarctica [104] and the potential applications of acetylated rice starch in the pharmaceutical and cosmetics industries. Xu et al. [105] studied the emulsion stabilized using octenylsuccinic anhydride-modified waxy maize starch. Here, the octenylsuccinic reaction of waxy maize starch was performed using β-amylase to increase its emulsification properties. Enzymatically modified starch is mainly used in the pharmaceutical industry owing to the unique property of releasing drugs at a constant rate [106]. Dura and Rosell [107] obtained porous starch using cyclodextrin glycosyltransferase enzyme (CGT). The authors reported that CGT improved the hydrolysis of the amorphous part of the starch, making more starch susceptible to enzymatic hydrolysis.

#### 2.5.2. Applications of Physically Modified Starch

Physically modified starch is also utilized in food and non-food applications. Agama-Acevedo et al. [108] and Chi et al. [109] prepared slowly digestible starch using HMT and dry heating treatment (DHT), respectively. Oat starch was modified using an ultrasound treatment by Falsafi and coworkers [110]. The authors reported the improved amylose content, swelling power, solubility, water and oil holding capacity, retrogradation tendency of starch upon ultrasound treatment, beneficial for food applications. Lei et al. [111] studied the effect of DHT on the properties of maize starch. Their results showed a decrease in the gelatinization enthalpy and increased shear resistance as the DHT increases. Moreover, the authors also reported that modification of starch using the DHT would benefit the industry.

Apart from food applications, physically modified starch is extensively used in non-food applications such as packaging and 3D printing. For example, Bharti and coworkers [112] prepared modified mango kernel starch using HMT, which showed potential applications for producing biodegradable films. Maniglia et al. [113] investigated the use of DHT modified cassava starch for 3D printing. Increased oxidation and granular size and decreased water absorption index, crystallinity, and apparent viscosity peak of starch were observed upon DHT. Interestingly, DHT could also enhance the pasting properties, gel texture, and printability of cassava starch.

#### 2.5.3. Applications of Chemically Modified Starch

Chemically modified starch shows distinct properties over native starch and has been broadly explored in food packaging, pharmaceutical, water treatment, and agriculture applications [1,114]. Table 5 shows the different types of applications in chemical modification processes.

Chemically modified starch is mainly used in the food packaging industry due to its film-forming ability. For example, Peidayesh et al. [115] conducted a study to explore the stability of mechanical and chemical properties of thermoplastic starch (TPS) by dual crosslinking using epichlorohydrin. This work indicated the potential of crosslinked TPS for packaging applications. The research carried out by Sharma et al. [116] showed the improved tensile strength of crosslinked fava bean starch thin films. In addition, the crosslinked starch films exhibited lower moisture content, water solubility, and water vapour permeability. Mittal and coworkers prepared PVA (polyvinyl alcohol)/starch and cellulosic material barley husk (BH)-based composite films using the grafting technique. Authors reported the use of composite films in biodegradable packaging films to replace synthetic non-degradable polymers [117]. Moreover, Moreno et al. [118] prepared biodegradable active films using oxidized corn starch. In this study, starch oxidation was performed using sodium periodate, and the films showed enhanced tensile strength and oxygen and water vapour barrier capacities and reduced migration to acid media.

Hiremani et al. [119] prepared packaging materials that possessed high mechanical strength, low water vapor permeability (WVP), antioxidant properties, and biodegradability. In addition, the food compatibility test showed that the migration rates of films were below the overall migration limit.

Bio-based adhesives have gained much attention due to health and environmental concerns caused by petroleum-based adhesives. For example, Monroy et al. [120] and Chen et al. [121] prepared bio-adhesives using chemically modified starch. Recently, Monroy et al. [120] investigated the bio-based adhesives using cassava starch, citric acid, and polycarboxylic acid. Zia-ud-Din et al. [121] prepared the bio-based adhesive using corn starch-g-poly (vinyl acetate-*co*-butyl acrylate). This study showed an increase in thermal stability. Furthermore, Chen et al. [122] developed the grafted starch-based wood adhesive using polyvinyl alcohol and sodium dodecyl sulfate.

Chemically modified starch has also been applied in pharmaceutical industries as biodegradable coatings and drug delivery systems. Hydrophilic and hydrophobic carboxymethyl starch derivatives were used to develop drug delivery systems, specifically micro-particles, nanoparticles, and hydrogels, by Lemos et al. [123]. Quadrado and Fajardo [124] prepared carboxymethyl starch (CMS) microparticles from rice starch with a low amylose content of (6%). They reported that CMS followed an ideal Zero-order kinetics, favorable for a drug delivery system showing the ability of microparticles-based CMS in control drug release at a specific site of the gastrointestinal tract. Mora and coworkers reported the potential of chemically modified waxy corn as emulsifying and stabilizing agents in pharmaceutical applications [125]. Apart from that, Jiang et al. [126] prepared the microcapsules using acid hydrolyzed and carboxymethyl corn starch. The authors reported the use of microcapsules for controlled drug delivery. Chemically modified starch has also been considered in the agriculture industry as a fertilizer. For example, Ibrahim et al. [127] prepared the controlled-release nitrogen fertilizer using crosslinked corn starch with improved stability and mechanical strength. Jyothi et al. [128] prepared cassava starch-graft-polyacrylonitrile-coated urea fertilizer, and the results exhibited the N release in the range of 69.8–78.3%. Apart from the fertilizers, modified starch was used in the preparation of agricultural mulch films. Merino et al. [129] reported that oxidation of corn starch nanocomposites could improve water resistance in agricultural mulch films. In contrast, Chen and coworkers prepared liquid starch-based mulching materials (LSMM) by grafting polyacrylic acid (PAA) onto starch and then crosslinked with *N*, *N*′-methylene-bisacrylamide (MBA). This study demonstrated high relative hygroscopicity, water retention, and degradability of LSMM [130].

Moreover, chemically modified starch also acts as a superabsorbent. Starch-based superabsorbents have been considered in personal care products, agriculture, forestry, chemical industry, and drug delivery systems. For instance, Czarnecka and Nowaczyk [131] prepared starch hydrogels crosslinked with acrylic monomers as a superabsorbent. They revealed that the water absorption capacity of hydrogels was 0.9%. Dispat et al. [132] synthesized a superabsorbent polymer for agricultural applications using modified starch. The starch was modified using zinc oxide and tetraethyl orthosilicate and then graft-copolymerized with potassium acrylate monomer. The modified starch showed enhanced reusability, biodegradation, provided support as a soil conditioner, and aided against transient drought.

Recently, Soto and coworkers investigated the removal of cationic, methylene blue (MB), and anionic, methyl orange (MO) dyes present in water using succinylated starch. This study indicated that succinylated starch was superabsorbent material while being sensitive to changes in pH and the ionic strength of the medium. They also showed the removal of MB from an aqueous solution using succinylated starch [133].

Gunawardene et al. [134] displayed the effectiveness of dual-modified cassava starch in removing Pb^2+^ ions in aqueous solutions. During starch modification, tetraethylorthosilicate (TEOS) was used as the chemical modifying agent, whereas Pluronic 123 was used as the structure-directing agent. The modified starch exhibited a maximum adsorption capacity of 330.3 mg/g while performing a higher desorption efficiency of over 97%.

**Figure 8:**
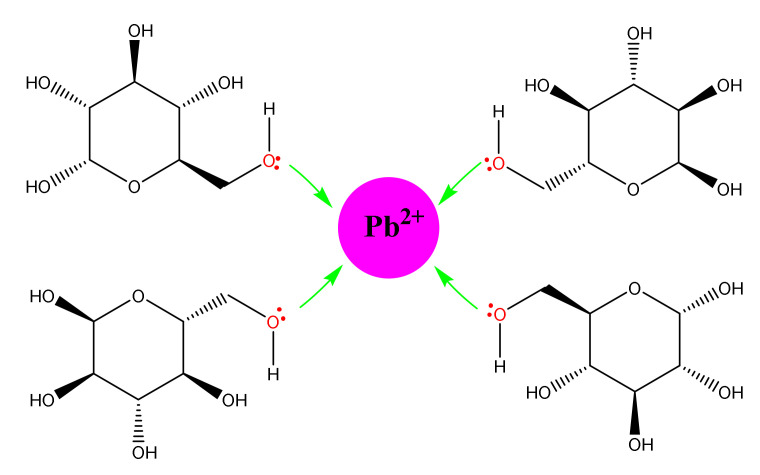
Pb^2+^ adsorption mechanism of modified cassava starch. Reprinted with permission from reference [134], Copyright 2021 MDPI.

Figure 8 shows the adsorption mechanism of Pb^2+^ ions onto modified starch. This adsorption behavior can be well explained using Pearson’s hard-soft acid-base (HSAB) principle. According to Figure 8, it is evident that the electron pairs in oxygen atoms in hydroxyl groups can donate electrons to the Pb^2+^, resulting in interactions between the lead ions and hydroxyl groups in modified cassava starch [134]. Table 5 summarizes the recent applications of modified starch relevant to packaging, pharmaceutical, agriculture, and sorption.

Apart from the above, Wang et al. [135] and Bai et al. [136] prepared an adsorbent for the removal of Cd^2+^ from aqueous solution using facile esterification reaction and graft copolymerization, respectively. Facile esterification reaction was used to prepare graphene-starch, and the results showed excellent adsorption towards Cd^2+^ ions at low concentrations from aqueous solutions [135]. Furthermore, Bai et al. [136] grafted amylose and amylopectin using sodium polyacrylate. According to the reported data, the starch-based superabsorbent showed 347.46 mg/g of monolayer adsorption capacity.

**Table 5:** Key applications of modified starch.

Applications	Modification Techniques	Examples	References
Packaging	Crosslinking	Cross-linked- TPS	[116]
		Cross-linked starch	[117]
		Cross-linked films of quaternary ammonium modified starch and Polyvinyl alcohol	[137]
	Grafting	PVA (Polyvinyl alcohol)/starch and cellulosic material barley husk (BH)	[117]
	Grafting	Polyvinyl alcohol/modifiedstarch-based biodegradablenanocomposite films	[138]
	Oxidation	7-Hydroxy-4-methylcoumarin doped Polyvinyl alcohol/oxidized maize starch	[119]
		Corn starch oxidation was performed using sodium periodate	[118]
	Dual Modification	Etherified–oxidized cassavastarch/Polyvinyl alcohol blends	[139]
Bio-based adhesives	Crosslinking	Cassava starch, citric acid and Polycarboxylic acid.	[120]
	Grafting	Corn starch-g-poly (vinyl acetate-*co*-butyl acrylate)	[121]
		Starch-g-polyvinyl alcohol	[122]
Pharmaceutical Industries	Etherification	Anionic carboxymethyl and cationic carboxymethyl	[140]
		Carboxymethyl starch	[141]
		Carboxymethyl starch	[123]
		Carboxymethyl starch	[124]
		Carboxymethyl corn starch	[126]
	Acetylation	Acetylated starch nano-crystals	[142]
	Crosslinking	Starch coated iron oxide nanoparticles	[143]
	Grafting	Fe_3_O_4_/starch-g-polyester nanocomposite	[144]
		Cellulose nanofiber-assisted starch graft-Polyacrylic acid	[145]
	Esterification	Octenyl succinic anhydride modified starch	[125]
Agriculture Industry	Crosslinking	Controlled-release nitrogen fertilizer	[127]
	Oxidization	Starch-Based Antibacterial Nanocomposites	[129]
	Grafting	Polyacrylic acid graft- starch	[130]
		Cassava starch-graft-polyacrylonitrile-coated urea fertilizer	[128]
Superabsorbents	Crosslinking	Starch crosslinked with acrylic monomers	[131]
		Crosslinked starch xanthate	[146]
		Dithiocarbamate-modified starch	[147]
		Fe_2_O_3_ nanoparticles-Starch nanocomposite	[148]
	Grafting	Modified starch with zinc oxide and tetraethyl orthosilicate and graft copolymerized with potassium acrylate monomer	[132]
		Low-density polyethylene-g-poly (acrylic 2 acid)-*co*-starch/organo-montmorillonite hydrogel	[149]
		Crown ether modification of starch	[150]
		Grafted amylose and amylopectin using poly(sodium acrylate)	[136]
	Esterification	Succinylated starch	[133]
		Facile esterification	[135]
		Modified corn starch with maleic acid (MA) and itaconic acid (IA)	[151]
	Dual modification	Modified cassava starch using tetraethylorthosilicate (TEOS) as the chemical modifying agent and Pluronic 123 as the structure directing agent	[134]
	Oxidations	Oxidized starch nanoparticles (SNPs)	[152]

## 3. Conclusions and Future Trends

Starch modification can be achieved by either genetic, enzymatic, physical, or chemical techniques. Although the enzymatic and genetic methods are not used in polymer industries due to the high production cost, they are considered mainly in the pharmaceutical and food industries. Physical methods are eco-friendly and low cost; however, they destroy starch structure limiting their industrial applications. Physically modified starch is used in food and non-food applications such as packaging and 3D printing. Chemical methods can alter the structural properties of starch to achieve the desired product attributes and are widely considered in the industry. Methods such as crosslinking, alkali treatment, etherification, esterification, acid hydrolyzing, grafting, and dual modifications can be used to prepare chemically modified starch and its derivatives. Chemical modification leads to changes in crystallinity, morphology, and molecular weight of starch while altering tensile strength, elongation at break, and adhesive properties of starch. Moreover, chemical modifications can also enhance the shelf-life and improve the quality, including excellent foaming, air, and moisture barrier properties. Chemically modified starch show potential in packaging, bio-based adhesives, pharmaceutical, agriculture, superabsorbent, and wastewater treatment applications. The major setbacks of modified starch include high water sensitivity, low mechanical properties, generation of toxic substances, and hard processability.

The modified starch market is expected to grow in the food and beverages industry, as emulsifying agents and stabilizing agents owing to its gluten-free nature. In addition, the high demand for processed food, increased use of bio-based adhesives in various industrial applications, and the increased interest in the development of starch-based products will contribute to modified starch market growth. Moreover, the increasing demand for natural sweeteners in the food and beverages industry and the use of modified starch in the paper, cosmetics, and pharmaceuticals will increase the global modified starch market in the future.

## Figures and Tables

**Table 1:** Main types of starch converting enzymes and their biological function.

Type of Starch Converting Enzymes	Biological Function	Example(s)	References
Endoamylses	Cleave the α-1,4-glucosidic bonds present in the inner part of the amylose or amylopectin chain	α-amylase	[39]
Exoamylases	Cleave both α-1,4 and α-1,6 bonds on amylose or amylopectin’s external glucose residues from the non-reducing end and produces only glucose (glucoamylase and α-glucosidases).	Glucoamylases α-glucosidases β-amylases	[40]
Debranching enzymes	Catalyze the hydrolysis of α-1,6-glucosidic bonds in amylopectin.	Amylo-1,6-glucosidase Pullulanase Isoamylase	[38]
Transferases	Cleave α-1,4-glucosidic bond of the donor molecule and transfer part of the donor to a glucosidic acceptor to form a new glucosidic bond.	Amylomaltase Cyclodextrin	[38]

**Table 2:** Enzymatic activity and optimum conditions of starch debranching enzymes.

Enzyme	Enzyme Activity (U)	Optimum Conditions	Substrate	Concentration of Enzyme (g) Required per 10 mL of Solution	References
Pullulanase EC 3.2.1.41	≥1000	pH 6.5 at 50 °C	Oligosaccharides Polysaccharides Pullulan	5	[38]
Isoamyalse EC 3.2.1.68	≥10,000,000	pH 6.5 at 40 °C	Polysaccharides	0.00050	[38]
Oligo-1,6-glucosidase EC 3.2.1.10	≥100	pH 6.8 at 37 °C	Polysaccharides	50	[38]

## Data Availability

Not applicable.
